# Aponermin or placebo in combination with thalidomide and dexamethasone in the treatment of relapsed or refractory multiple myeloma (CPT-MM301): a randomised, double-blinded, placebo-controlled, phase 3 trial

**DOI:** 10.1186/s12885-023-11489-8

**Published:** 2023-10-14

**Authors:** Zhongjun Xia, Yun Leng, Baijun Fang, Yang Liang, Wei Li, Chengcheng Fu, Linhua Yang, Xiaoyan Ke, Hua Jiang, Jianyu Weng, Li Liu, Yaozhong Zhao, Xuejun Zhang, Zhongxia Huang, Aichun Liu, Qingzhi Shi, Yuhuan Gao, Xiequn Chen, Ling Pan, Zhen Cai, Zhao Wang, Yafei Wang, Yaqun Fan, Ming Hou, Yigai Ma, Jianda Hu, Jing Liu, Jianfeng Zhou, Xiaohong Zhang, Haitao Meng, Xuzhang Lu, Fei Li, Hanyun Ren, Bintao Huang, Zonghong Shao, Hebing Zhou, Yu Hu, Shifang Yang, Xiangjun Zheng, Peng Wei, Hongyan Pang, Wei Yu, Yuzhang Liu, Sujun Gao, Lingzhi Yan, Yanping Ma, Hongmei Jing, Juan Du, Wei Ling, Jingyi Zhang, Weiwei Sui, Fuxu Wang, Xin Li, Wenming Chen

**Affiliations:** 1 Department of Hematologic Oncology, Sun Yat-Sen University Cancer Center, Guangzhou, China; 2 Department of Hematology, Beijing Chao-Yang Hospital Capital Medical University, Beijing, China; 3 Department of Hematology, Henan Cancer Hospital, Henan Cancer Hospital Affiliated to Zhengzhou University, Zhengzhou, China; 4 Department of Hematology, The First Hospital of Jilin University, Changchun, China; 5 Department of Hematology, The First Affiliated Hospital of Soochow University, Jiangsu Institute of Hematology National Clinical Research Center for Hematologic Diseases, Suzhou, China; 6 Department of Hematology, Second Hospital of Shanxi Medical University, Taiyuan, China; 7 Department of Hematology and Lymphoma Research Center, Peking University Third Hospital, Beijing, China; 8 Department of Hematology, Changzheng Hospital, Second Military Medical University, Shanghai, China; 9 Department of Hematology, Guangdong Provincial People’s Hospital, Guangdong Academy of Medical Sciences, Guangzhou, China; 10 Department of Hematology, Tangdu Hospital, Fourth Military Medical University, Xi’an, China; 11 Institute of Hematology & Blood Diseases Hospital, Chinese Academy of Medical Sciences & Peking Union Medical College, Tianjin, China; 12 Department of Hematology, The Second Hospital of Hebei Medical University, Shijiazhuang, China; 13 Department of Hematology, Harbin Medical University Cancer Hospital, Harbin, China; 14 Department of Hematology, The Second Affiliated Hospital of Nanchang University, Nanchang, China; 15 Department of Hematology, Fourth Hospital of Hebei Medical University, Shijiazhuang, China; 16 Department of Hematology, XiJing Hospital, Fourth Military Medical University, Xi’an, China; 17 Department of Hematology, West China Hospital, Sichuan University, Chengdu, China; 18 Bone Marrow Transplantation Center, The First Affiliated Hospital, School of Medicine, Zhejiang University, Hangzhou, China; 19 Department of Hematology, Beijing Friendship Hospital, Capital Medical University, Beijing, China; 20 Department of Hematology, Tianjin Medical University Cancer Institute and Hospital, Tianjin, China; 21 Department of Hematology, The First Affiliated Hospital of Xiamen University and Institute of Hematology, Medical College of Xiamen University, Xiamen, China; 22 Department of Hematology, Qilu Hospital of Shandong University, Jinan, China; 23 Department of Hematology, China-Japan Friendship Hospital, Beijing, China; 24 Fujian Medical University Union Hospital, Fujian Institute of Hematology, Fujian Province Key Laboratory of Hematology, Fuzhou, China; 25 Department of Hematology, The Third Xiangya Hospital of Central South University, Changsha, China; 26 Department of Hematology, Tongji Hospital of Tongji Medical College, Huazhong University of Science and Technology, Wuhan, China; 27 Department of Hematology, The Second Affiliated Hospital of Zhejiang University School of Medicine, Hangzhou, China; 28 Department of Hematology, The First Affiliated Hospital, School of Medicine, Zhejiang University, Hangzhou, China; 29 Department of Hematology, The Affiliated Changzhou No.2 People’s Hospital of Nanjing Medical University, Changzhou, China; 30 Department of Hematology, First Affiliated Hospital of Nanchang University, Nanchang, China; 31 Department of Hematology, Peking University First Hospital, Beijing, China; 32 Department of Hematology, The Affiliated Hospital of Inner Mongolia Medical University, Hohhot, China; 33 Department of Hematology, General Hospital of Tianjin Medical University, Tianjin, China; 34 Department of Hematology, Beijing Luhe Hospital, Capital Medical University, Beijing, China; 35 Department of Hematology, Union Hospital, Tongji Medical College, Huazhong University of Science and Technology, Wunan, China; 36 Beijing Sunbio Biotech Co., Ltd., Beijing, China

**Keywords:** Aponermin, TNF-related apoptosis-inducing ligand, Multiple myeloma, Relapsed/refractory, Phase 3

## Abstract

**Background:**

Aponermin, a circularly permuted tumor necrosis factor-related apoptosis-inducing ligand, is a potential death receptor 4/5-targeted antitumour candidate. Previous phase 1/2 studies have demonstrated the efficacy of aponermin in patients with relapsed or refractory multiple myeloma (RRMM). To confirm the superiority of aponermin plus thalidomide and dexamethasone (aponermin group) over placebo plus thalidomide and dexamethasone (placebo group) in RRMM, a randomized, double-blinded, placebo controlled phase 3 trial was performed.

**Methods:**

Four hundred seventeen patients with RRMM who had previously received at least two regimens were randomly assigned (2:1) to receive aponermin, thalidomide, and dexamethasone or placebo, thalidomide, and dexamethasone. The primary endpoint was progression-free survival (PFS). Key secondary endpoints included overall survival (OS) and overall response rate (ORR).

**Results:**

A total of 415 patients received at least one dose of trial treatment (276 vs. 139). The median PFS was 5.5 months in the aponermin group and 3.1 months in the placebo group (hazard ratio, 0.62; 95% confidence interval [CI], 0.49–0.78; *P* < 0.001). The median OS was 22.4 months for the aponermin group and 16.4 months for the placebo group (hazard ratio, 0.70; 95% CI, 0.55–0.89; *P* = 0.003). Significantly higher rates of ORR (30.4% vs. 13.7%, *P* < 0.001) and very good partial response or better (14.1% vs. 2.2%, *P* < 0.0001) were achieved in the aponermin group than in the placebo group. Treatment with aponermin caused hepatotoxicity in some patients, as indicated by the elevated alanine transaminase, aspartate transaminase, or lactate dehydrogenase levels (52.2% vs. 24.5%, 51.1% vs. 19.4% and 44.9% vs. 21.6%, respectively), mostly grade 1/2, transient and reversible. The main grade 3/4 adverse events included neutropenia, pneumonia and hyperglycemia. The incidence of serious adverse events was similar between the two groups (40.6% vs. 37.4%). There was no evidence that aponermin leads to hematological toxicity, nephrotoxicity, cardiotoxicity, or secondary tumors.

**Conclusions:**

Aponermin plus thalidomide and dexamethasone significantly improved PFS, OS and ORR with manageable side effects in RRMM patients who had received at least two prior therapies. These results support the use of aponermin, thalidomide, and dexamethasone as a treatment option for RRMM patients.

**Trial registration:**

The trial was registered at http://www.chictr.org.cn as ChiCTR-IPR-15006024, 17/11/2014.

**Supplementary Information:**

The online version contains supplementary material available at 10.1186/s12885-023-11489-8.

## Introduction

Although in the past 20 years, several new drugs have been approved, multiple myeloma (MM) remains an incurable hematological malignancy, and almost all patients eventually become drug-resistant [1–4]. New drugs are critically needed.

Tumor necrosis factor-related apoptosis-inducing ligand (TRAIL) induces apoptosis selectively by activating death receptor 4 or 5 (DR4/5) in a wide range of cancers while sparing normal cells [5–7]. Several recombinant TRAIL-fusion proteins and multimeric anti-DR5 agonist antibody are in clinical trials for cancers [8–13].

Aponermin is a recombinant circularly permuted human TRAIL (CPT), by connecting the amino end and the carboxy end of the native TRAIL fragment (amino acid 121–281) with the linker (Gly-Gly-Gly-Gly-Gly) and breaking the TRAIL fragment at the site of amino acid 135 to create new amino and carboxy termini. The primary sequence of the aponermin protein was reordered, while its secondary structure and activity were retained. Aponermin is a more stable homotrimer and has shown a higher affinity for DR4/5, more potent antitumor activity and longer half-life than native TRAIL [14–17].

In the phase 1b study in patients with relapsed or refractory multiple myeloma (RRMM) [18], aponermin monotherapy was well tolerated with doses ranging 5–15 mg/kg. An overall response rate (ORR) of 18.5% was achieved across dose ranges. In the phase 2 study of aponermin combined with thalidomide in RRMM patients [19], a higher ORR (22.0% vs. 16.7%) and more cases of complete response (CR) or near CR (12.2% vs. 0) were observed compared to the aforementioned phase 1b results [18] at the same dose level, even though the patients were more heavily pretreated in the phase 2 trial.

Thalidomide combined with dexamethasone (TD regimen) has been approved for the treatment of MM in 2006. Although TD regimen is no longer widely used in developed countries with the approval of novel drugs, it is still a good option in low- and middle-income countries due to its accessibility and affordability [20, 21]. Preclinical studies in xenografted nude mice of human multiple myeloma showed that the antitumor effect of aponermin combined with TD was significantly better than that of aponermin alone or TD alone (*P* < 0.05) (data not published). In a randomized, open-labelled phase 2 trial [22], a prolonged progression-free survival (PFS) (6.7 months) was observed in the aponermin plus TD group compared to that of the TD group (3.1 months). A higher ORR and clinical benefit rate were also observed. To confirm the superiority of aponermin plus TD over placebo plus TD in patients with RRMM, a phase 3 trial (CPT-MM301) was performed.

## Methods

### Trial design

CPT-MM301 was a multicentre, randomized, double-blinded, placebo-controlled phase 3 trial conducted in China. The trial protocol was designed by sponsors and investigators, and was approved by the independent ethics committees of Beijing Chao-Yang Hospital Capital Medical University. The trial was conducted in accordance with the principles of Good Clinical Practice and the Declaration of Helsinki. All patients provided written informed consent before enrolment. The data were collected, analysed, and interpreted by investigators and sponsor. Investigators had full accessibility to all data. The trial was registered at http://www.chictr.org.cn as ChiCTR-IPR-15006024, 17/11/2014.

### Patients

Eligible participants were enrolled in this study. Key inclusion criteria were as follows: participants' age: 18–75 years; previous treatment with two or more regimens for MM and not considered for bone marrow transplantation; M-protein levels needed to meet at least one of the following criteria: serum M-protein ≥ 10 g/L (IgG, IgM, IgD subtype), or ≥ 7.5 g/L (IgA subtype), or urinary M-protein ≥ 200 mg/24 h; absolute neutrophil count ≥ 1.0 × 10^9^/L; platelet ≥ 50 × 10^9^/L; aspartate transaminase (AST) ≤ 2.5 × upper limit of normal (ULN); alanine transaminase (ALT) ≤ 2.5 × ULN; alkaline phosphatase ≤ 2.5 × ULN; total bilirubin (TBIL) ≤ 1.5 × ULN; creatinine clearance rate ≥ 30 ml/min. Key exclusion criteria included: refractoriness to TD or lenalidomide plus dexamethasone (RD) regimens of the last treatment; received any anti-MM drug treatment within 4 weeks before the trial; participated in aponermin clinical trials previously; had serious organic or mental diseases. (see Supplementary file).

### Randomization and masking

An allocation ratio preserving biases coin randomization was used in this trial (block size of 6) [23]. The random allocation sequence was generated using SAS 9.2 and uploaded to an interactive web response system (IWRS) by an unblinded system administrator. Eligible patients were enrolled and randomly assigned (2:1) by investigators via the IWRS to receive either aponermin plus TD (aponermin group), or placebo plus TD (placebo group). Randomization was stratified according to the number of prior therapeutic regimens (≤ 3 or > 3), the status of TD/RD therapy (yes vs. no), and International Staging System (ISS) stage (stage I vs. stage II or III). Aponermin and placebo were packaged in a blinded manner under the supervision of a statistician according to the drug list. The packaging and labels of aponermin and placebo were identical to ensure that they remained masked to the treatment assignment. The investigators, participants, research staff, members of the independent assessment committee (IAC), and sponsor study team were masked to the treatment location.

### Procedures

In each cycle, patients were administered 10 mg/kg of aponermin or placebo via intravenous infusion on days 1–5, oral thalidomide 150 mg on days 1–28, and oral dexamethasone 40 mg on days 1–4 for 18 cycles (28 days for each cycle). In the first cycle, thalidomide was administered on days 2–28, and dexamethasone on days 2–5 to observe the changes in AST, ALT, and lactate dehydrogenase (LDH) after the first dose of aponermin alone. Treatment was continued for up to 18 cycles or until progressive disease (PD), unacceptable toxicities, or withdrawal from the study. After completing 18 cycles of treatment, patients might receive further treatment based on the investigators' opinions.

### Outcomes and assessments

The primary endpoint was PFS, defined as the time from the date of the randomization to the date of the first documented PD or death from any cause during the study, whichever occurred first. Secondary endpoints included overall survival (OS), ORR, duration of response (DOR), time to response (TTR), time to progression (TTP), safety, and health-related quality of life (HRQoL). The exploratory endpoint was to evaluate the efficacy among high-risk patients defined as [t(14;16)], [t(4;14)], or [del(17p)] by fluorescence in situ hybridization, or chromosome 13 deletion with hypodiploidy by G-band staining [24].

Serum and urine monoclonal proteins and serum free light chain levels were measured at a central laboratory. Disease status were assessed by the investigators at the baseline and the end of each cycle. The International Myeloma Working Group consensus criteria (IMWG criteria) were used to assess responses and PD. For patients in remission, if treatment was discontinued due to intolerable adverse event (AE) or the completion of 18 cycles of treatment, the disease status was assessed every six weeks until PD, death or next anti-myeloma therapy started. We required all responses and PD be assessed by IAC. PFS, ORR, DOR, TTR, and TTP were calculated based on the responses and PD assessed by IAC.

The HRQoL questionnaires were completed at baseline and the end of every cycle using the European Organization for Research and Treatment of Cancer (EORTC) questionnaires: the generic EORTC QLQ-C30 and the myeloma-specific QLQ-MY20.

The AEs and serious adverse events (SAEs) were collected up to 28 days following the last treatment dose and graded according to the National Cancer Institute Common Terminology Criteria for Adverse Events Version 4.03.

### Statistical analysis

The sample size was determined based on a conservative estimation of the median PFS of 5.0 months in the aponermin group and 3.5 months in the placebo group. According to ethical opinion, the proportion of patients in the aponermin and placebo groups was 2:1. It was estimated that a total of 286 events of PD or death over a 30-months enrollment period and a 6-months follow-up would be required to have a statistical power of 80%. This is to show superiority at a hazard ratio (HR) of 0.70 using a log-rank test (one-sided alpha is 0.025). A total of 313 patients were needed based on the calculation before factoring in the dropout rate. However, considering a 25% dropout rate, at least 417 patients were required (278 in the aponermin group and 139 in the placebo group).

No interim analyse was done in this trial. A final analysis was performed when the last participant had been enrolled for 6 months.

In this study, all efficacy analyses were based on the modified intention-to-treat population (mITT), including all randomized patients who received at least one dose of the trial treatment. The primary endpoint, PFS, was compared using a prespecified stratified log-rank test. The Kaplan–Meier method was used to estimate the median PFS and depict the curve. The 95% confidence interval (CI) was estimated using the Brookmeyer-Crowley formula. HR and 95% CI were estimated using a stratified Cox proportional hazards model. The proportional hazards assumption was assessed and met by the Cox regression model, using time-dependent explanatory variables. The aforementioned strata variables were the same as those used in the randomization. Other time-to-event data, including OS, DOR, TTR, TTP, and subgroup analyses of PFS and OS, were analysed using a method similar to PFS. The ORR, clinical benefit rate and the rate of each response were compared between the groups using chi-squared tests. The Clopper–Pearson method was used to calculate the 95% CI. Subgroup analyses of ORR were performed using similar method. Scores for the EORTC QLQ-30 and MY20 were calculated according to the developer's scoring manual. The raw scores from the scales in both questionnaires were standardized by linear transformation to range 0–100. Descriptive statistics for the baseline scores for each domain were summarized, and the differences between groups were assessed using a group t-test. A mixed-model measure analysis was used to estimate the treatment effects over time for each domain (longitudinal analysis) and assess the differences between groups. The safety analysis included all patients who received at least one dose of the trial treatment. AEs were coded using MedDRA version 22.1. The frequency of AEs was reported.

All statistical analyses were conducted using SAS software (version 9.4). Two-sided *P*-values < 0.05 were considered statistically significant.

## Results

### Patients

**Fig. 1:**
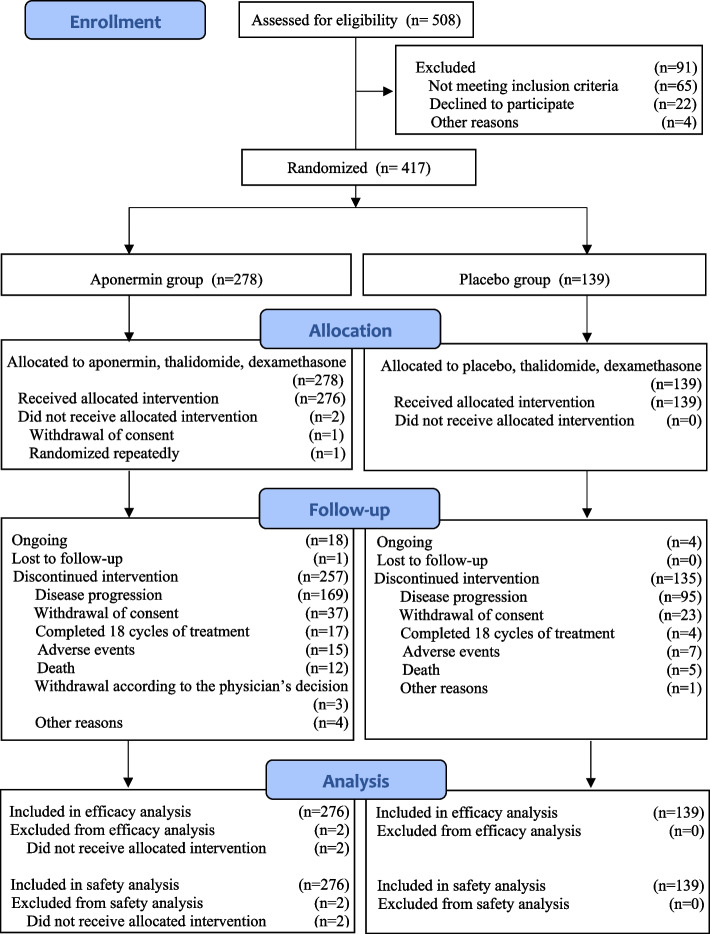
CONSORT flow diagram

From February 25, 2015, to July 3, 2019, 508 patients were screened in 36 hospitals in China. A total of 417 patients were eligible and randomly assigned (2:1) to the aponermin group (278 patients) or the placebo group (139 patients). Of these, 276 patients in the aponermin group and 139 in the placebo group received the study treatment and were included in the analysis of efficacy and safety. By the final analysis date (January 3, 2020), 257 (93.1%) patients in the aponermin group and 135 (97.0%) patients in the placebo group had discontinued treatment. The reasons for the discontinuation of the intervention are shown in Fig. 1. The median treatment duration for the aponermin group was significantly longer than that of the placebo group (5 vs. 3 cycles).

The baseline characteristics were well balanced between the two groups (Table 1). The median age was 59 years (range, 26–75), 42.2% were women. The median time since the initial diagnosis of MM was 2.6 years (range, 0.2–14.7). The median number of prior treatment regimens was three (range, 2–25), of which 46.0% had received at least four regimens. All patients had received previous glucocorticoids; 73.6%, proteasome inhibitor (PI); and 86.6%, immunomodulator (IMiD).

**Table 1:** Baseline characteristics of the intention-to-treat population

	**Aponermin Group (*N* = 278)**	**Placebo Group (*N* = 139)**	**Overall (*N* = 417)**	***P-*value**
Age				
Median (range) — yr	59.0 (26–75)	59.0 (33–75)	59.0 (26–75)	0.29
< 65 yr — no. (%)	207 (74.5)	107 (77.0)	314 (75.3)	0.57
≥ 65 yr — no. (%)	71 (25.5)	32 (23.0)	103 (24.7)	
Female sex — no. (%)	121 (43.5)	55 (39.6)	176 (42.2)	0.44
Creatinine clearance rate ≥ 60 ml/min — no. (%)	228 (82.0)	110 (79.1)	338 (81.1)	0.48
International Staging System stage — no. (%)				0.15
I	98 (35.3)	47 (33.8)	145 (34.8)	
II	112 (40.3)	46 (33.1)	158 (37.9)	
III	68 (24.5)	46 (33.1)	114 (27.3)	
Median time since initial diagnosis (range) — yr	2.65 (0.2–14.7)	2.60 (0.3–13.5)	2.60 (0.2–14.7)	0.74
Median no. of prior treatment regimens (range)	3 (2–25)	3 (2–18)	3 (2–25)	0.51
≥ 4 — no. (%)	130 (46.8)	62 (44.6)	192 (46.0)	0.68
Previous therapy — no. (%)				
Glucocorticoid	278 (100)	139 (100)	417 (100)	
Thalidomide/lenalidomide and dexamethasone	207 (74.5)	104 (74.8)	311 (74.6)	0.94
Immunomodulator	246 (88.5)	115 (82.7)	361 (86.6)	0.10
Lenalidomide	83 (29.9)	32 (23.0)	115 (27.6)	0.14
Thalidomide	220 (79.7)	103 (74.1)	323 (77.8)	0.18
Proteasome inhibitor	206 (74.1)	101 (72.7)	307 (73.6)	0.75
Immunomodulator and proteasome inhibitor	181 (65.1)	83 (59.7)	264 (63.3)	0.28
Stem-cell transplantation	48 (17.3)	28 (20.1)	76 (18.2)	0.47
Refractory multiple myeloma — no. (%)	97 (34.9)	48 (34.5)	145 (34.8)	0.94
Refractory to immunomodulator and proteasome inhibitor — no. (%)	41 (14.7)	13 (9.4)	54 (12.9)	0.12
Refractory to immunomodulator — no. (%)	125(45.0)	47(33.8)	172(41.2)	0.03
Cytogenetic profile — no. (%)^a^				
Standard risk	138/201 (68.7)	64/98 (65.3)	202/299 (67.6)	0.56
High risk	63/201 (31.3)	34/98 (34.7)	97/299 (32.4)	
Extramedullary disease of multiple myeloma	21(7.6)	10(7.2)	31(7.4)	0.90

There were no significant differences at baseline between the two groups in the characteristics shown

^a^High-risk cytogenetic abnormalities were detected by karyotype analysis and fluorescence in situ hybridization (FISH) analysis, and were defined as chromosome 13 deletion with hypodiploid, chromosome 17p deletion [del(17p)], translocation between chromosomes 14 and 16 [t(14;16)], or translocation between chromosomes 4 and 14 [t(4;14)]

### Efficacy

**Fig. 2:**
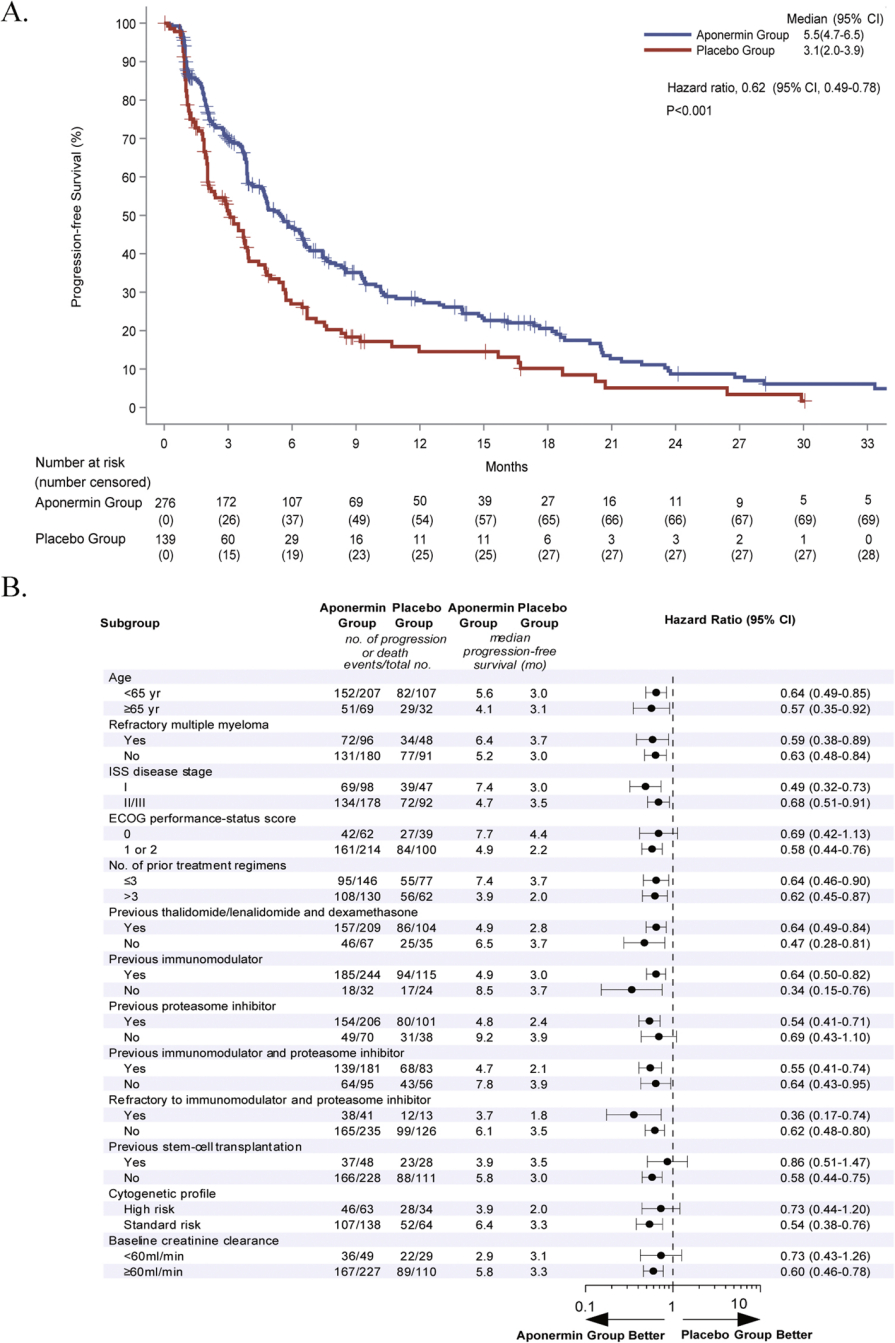
Progression-free Survival. **A** Kaplan–Meier analysis of progression-free survival (response assessed by Independent Assessment Committee) in the modified Intention-to-Treat Population, which included all patients who received at least one dose of trial treatment. **B** Subgroup analysis of progression-free survival

At a median follow-up of 17.2 months (95% CI, 15.1–28.2), 203 (73.6%) events of PD or death occurred in the aponermin group and 111 (79.9%) in the placebo group as assessed by the IAC. The median PFS was 5.5 months (95% CI, 4.7–6.5) in the aponermin group vs. 3.1 months (95% CI, 2.0–3.9) in the placebo group (HR, 0.62; 95% CI, 0.40–0.78; *P* < 0.001) (Fig. 2A). A significantly prolonged PFS was also observed based on the investigator's evaluations (Supplementary file: Table S1).

The prespecified subgroup analysis showed that the effect of aponermin on prolonging PFS compared to placebo was consistent for most subgroups (Fig. 2B). The PFS benefits of the aponermin group vs. placebo group were particularly evident in the subgroups of the patients with refractory MM (median 6.4 vs. 3.7 months; HR, 0.59; 95% CI, 0.38–0.89), the patients with prior therapy of IMiD and PI (median 4.7 vs. 2.1 months; HR, 0.55; 95% CI, 0.41–0.74), and the patients who were refractory to both IMiD and PI (median 3.7 vs. 1.8 months; HR, 0.36; 95% CI, 0.17–0.74).

**Fig. 3:**
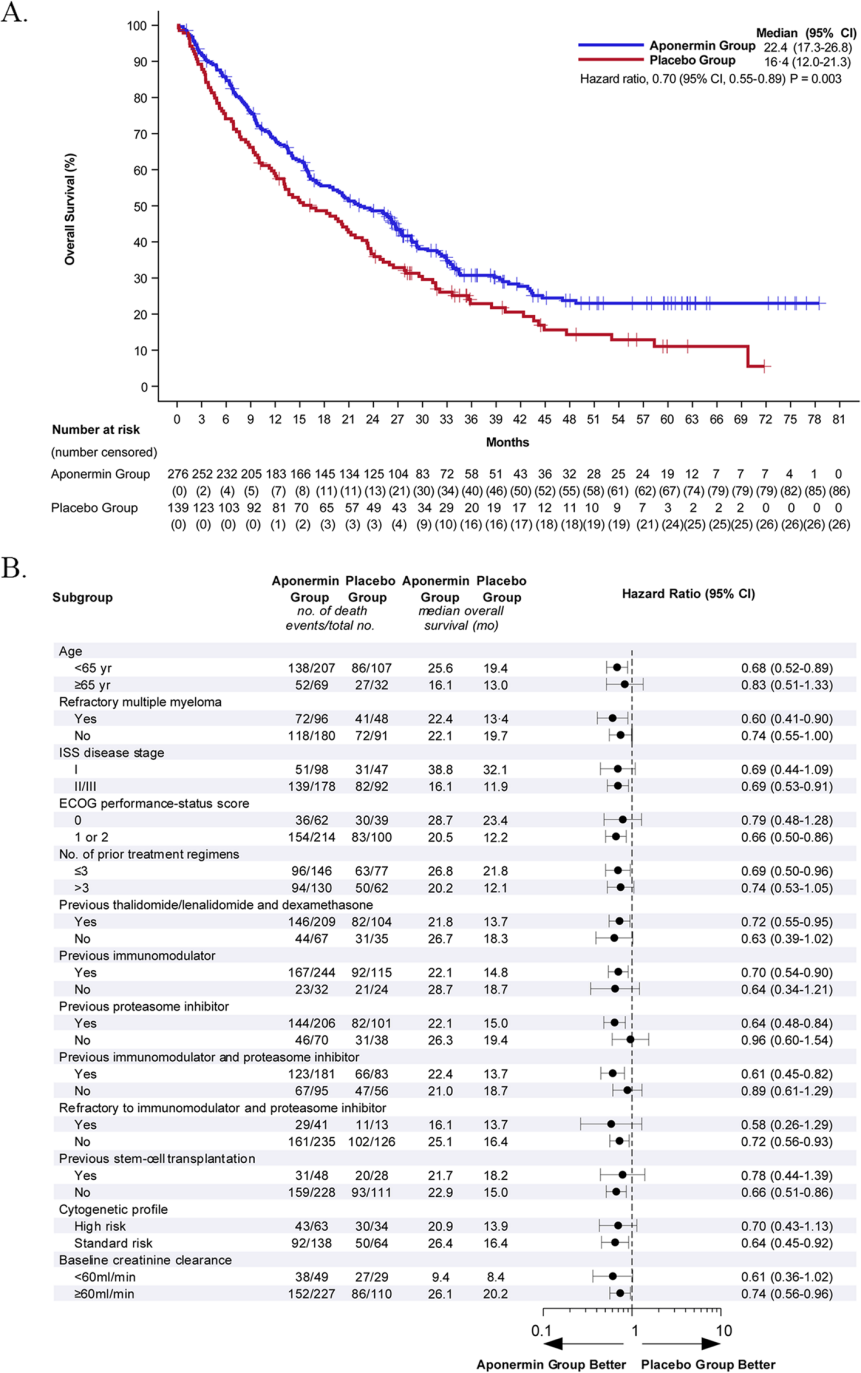
Overall Survival. **A**. Kaplan–Meier analysis of overall survival in the modified Intention-to-Treat Population, which included all patients who received at least one dose of trial treatment. **B** Subgroup analysis of overall survival

At a median follow-up of 30.1 months (95% CI, 25.9–34.0), 151 (54.7%) deaths occurred in the aponermin group, and 88 (63.3%) in the placebo group. The median OS was 21.8 months (95% CI, 17.3–27.3) in the aponermin group and 17.0 months (95% CI, 12.2–23.2) in the placebo group (HR, 0.72; 95% CI, 0.56–0.94; *P* = 0.02) (Supplementary file: Figure S1). In an updated analysis of OS with a median follow-up of 48.0 months (95% CI, 40.0–55.7), 6.0 months extension was observed in the aponermin group compared to that in the placebo group (median, 22.4 vs. 16.4 months; HR, 0.70; 95% CI, 0.55–0.89; *P* = 0.003) (Fig. 3A). The prespecified subgroup analysis showed a significant effect of the aponermin group compared with the placebo group on OS for most of the subgroups (Fig. 3B).

The median DOR was 15.2 months (95% CI, 10.0–18.2) in the aponermin group vs. 9.8 months (95% CI, 4.4–14.7) in the placebo group (HR, 0.55; 95% CI, 0.29–1.05; *P* = 0.07). The median TTR were both 1.9 months in the two groups (HR, 0.89; 95% CI, 0.53–1.51; *P* = 0.67). TTP was longer in the aponermin group than in the placebo group (median, 5.8 vs. 3.5 months; HR, 0.61; 95% CI, 0.48–0.78; *P* < 0.001) (Table 2).

**Table 2:** The efficacy results assessed by independent assessment committee in modified intention-to-treat population

	**Aponermin Group (*N* = 276)**	**Placebo Group (*N* = 139)**	**Proportion difference between groups**	**Hazard Ratio (95%CI)**	***P*-value**
Overall response —% (95% CI)	30.4 (25.1–36.2)	13.7 (8.4–20.5)	16.8 (8.9–24.6)	··	< 0.001
Clinical benefit — % (95% CI)^a^	45.3 (39.3–51.4)	29.5 (22.1–37.8)	15.8 (6.2–25.4)	··	0.002
Very good partial response or better— % (95% CI)	14.1 (10.2–18.8)	2.2 (0.4–6.2)	12.0 (7.2–16.7)	··	< 0.001
Best response — % (95% CI)^b^					
Stringent complete response	0 (0–1.3)	0 (0–2.6)	··	··	··
Complete response	2.2 (0.8–4.7)	0.7 (0.0–3.9)	1.5 (-0.8–3.7)	··	0.43
Very good partial response	12.0 (8.4–16.4)	1.4 (0.2–5.1)	10.5 (6.2–14.8)	··	< 0.001
Partial response	16.3 (12.1–21.2)	11.5 (6.7–18.0)	4.8 (-2.1–11.7)	··	0.24
Minimal response^c^	14.9 (10.9–19.6)	15.8 (10.2–23.0)	-1.0 (-8.3–6.4)	··	0.77
Stable disease	38.0 (32.3–44.1)	44.6 (36.2–53.3)	-6.6 (-16.6–3.5)	··	0.21
Progression	12.3 (8.7–16.8)	23.7 (16.9–31.7)	-11.4 (-19.5– -3.4)	··	0.004
Not evaluable	4.3 (2.3–7.5)	2.2 (0.4–6.2)	2.2 (-1.2–5.6)	··	0.40
Median time to response — mo (95% CI)	1.9 (1.2–1.9)	1.9 (1.0–2.6)	··	0.89 (0.53–1.51)	0.67
Median duration of response — mo (95% CI)	15.2 (10.0–18.2)	9.8 (4.4–14.7)	··	0.55 (0.29–1.05)	0.07
Median time to progression — mo (95% CI)	5.8 (4.8–7.4)	3.5 (2.1–4.4)	··	0.61 (0.48–0.78)	< 0.001

^a^A clinical benefit was defined as a minimal response or better

^b^The best confirmed responses were assessed by the independent assessment committee in a blinded manner according to the International Myeloma Working Group criteria

^c^Minimal response was assessed according to the European Group for Blood and Bone Marrow Transplant criteria

According to the assessment of the IAC, the ORR was 30.4% (95% CI, 25.1%–36.2%) in the aponermin group vs. 13.7% (95% CI, 8.4%–20.5%) in the placebo group (*P* < 0.001). The rates of very good partial response (VGPR) or better (14.1% vs. 2.2%), VGPR (12.0% vs. 1.4%), and clinical benefit (MR or better) (45.3% vs. 29.5%) were all superior in the aponermin group than those in the placebo group (Table 2). In a prespecified subgroup for patients who had achieved PR or better responses, greater benefits in PFS (median, 17.6 vs. 10.7 months; HR, 0.584; 95% CI, 0.31–1.11; *P* = 0.10) and OS (median, 42.9 vs. 31.6 months; HR, 0.41; 95% CI, 0.18–0.92; *P* = 0.03) were observed. The ORR and other response rates assessed by the investigators were similar to those assessed by the IAC (Supplementary file: Table S1).

### Safety

AEs that occurred in 15% or more of the patients in either group are shown in Table 3. The main grade 3 or 4 AEs included neutropenia (26.8% vs. 26.6%), pneumonia (25.0% vs. 23.7%), and hyperglycemia (21.0% vs. 12.2%) in the aponermin and placebo groups. Hepatotoxicity occurred at a significantly higher frequency in the aponermin group than in the placebo group (ALT, 52.2% vs. 24.5%; AST, 51.1% vs. 19.4%; LDH, 44.9% vs. 21.6%). However, most of the ALT and AST elevations were grade 1 to 2, and approximately 10% of patients exhibited grade 3 or 4 elevations in the aponermin group. All hepatotoxicity events were transient and returned to normal or baseline levels before the next treatment. The incidence of dose adjustment or discontinuation due to hepatotoxicity was < 3%. No case of liver failure or death due to aponermin-related hepatotoxicity was reported.

**Table 3:** Adverse events

	**Aponermin Group (*N* = 276)**		**Placebo Group (*N* = 139)**		***P-*value** ***P***_**1**_**, *P***_**2**_
	**Any grade**	**Grade 3 or 4**	**Any grade**	**Grade 3 or 4**	
**Nonhematologic adverse events**					
Hyperglycemia	235 (85.1)	58 (21.0)	115 (82.7)	17 (12.2)	0.52, 0.03
Increased alanine aminotransferase	145 (52.5)	26 (9.4)	34 (24.5)	1 (0.7)	< 0.001, < 0.001
Increased aspartate aminotransferase	141 (51.1)	33 (12.0)	27 (19.4)	1 (0.7)	< 0.001, < 0.001
Hypocalcemia	129 (46.7)	15 (5.4)	48 (34.5)	3 (2.2)	0.02, 0.12
Hypokalemia	129 (46.7)	42 (15.2)	60 (43.2)	14 (10.1)	0.49, 0.15
Increased lactate dehydrogenase	124 (44.9)	16 (5.8)	30 (21.6)	0	< 0.001, 0.004
Constipation	118 (42.8)	8 (2.9)	64 (46.0)	1 (0.7)	0.52, 0.15
Urine sugar detected	113 (40.9)	11 (4.0)	56 (40.3)	7 (5.0)	0.90, 0.62
Hypoalbuminemia	110 (39.9)	11 (4.0)	68 (48.9)	4 (2.9)	0.08, 0.57
Infectious pneumonia	102 (37.0)	69 (25.0)	53 (38.1)	33 (23.7)	0.82, 0.78
Fatigue	100 (36.2)	6 (2.2)	43 (30.9)	1 (0.7)	0.28, 0.28
Upper respiratory tract infection	76 (27.5)	21 (7.6)	24 (17.3)	7 (5.0)	0.02, 0.32
Increased blood urea	70 (25.4)	3 (1.1)	33 (23.7)	1 (0.7)	0.72, 0.72
Proteinuria/urinary protein detected	87 (31.5)	1 (0.4)	34 (24.5)	1 (0.7)	0.14, 0.62
Hypophosphatemia	67 (24.3)	17 (6.2)	28 (20.1)	13 (9.4)	0.34, 0.24
Hyperuricemia	65 (23.6)	19 (6.9)	19 (13.7)	7 (5.0)	0.02, 0.46
Dizzy	58 (21.0)	0	25 (18.0)	0	0.47, NA
Pyrexia	57 (20.7)	0	17 (12.2)	0	0.03, NA
Hyponatremia	53 (19.2)	22 (8.0)	28 (20.1)	13 (9.4)	0.82, 0.63
Hypertriglyceridemia	53 (19.2)	5 (1.8)	16 (11.5)	1 (0.7)	0.05, 0.38
Peripheral edema	53 (19.2)	2 (0.7)	20 (14.4)	1 (0.7)	0.22, 1.00
Hypesthesia	52 (18.8)	5 (1.8)	24 (17.3)	1 (0.7)	0.70, 0.38
Positive urine leukocyte	44 (15.9)	1 (0.4)	12 (8.6)	0	0.04, 0.48
Diarrhea	44 (15.9)	1 (0.4)	17 (12.2)	2 (1.4)	0.31, 0.22
Sinus bradycardia	42 (15.2)	1 (0.4)	19 (13.7)	0	0.67, 0.48
Drowsiness	38 (13.8)	0	25 (18.0)	0	0.26, NA
Increased blood creatinine	37 (13.4)	6 (2.2)	21 (15.1)	5 (3.6)	0.64, 0.39
**Hematologic adverse events**					
Neutropenia	140 (50.7)	74 (26.8)	75 (54.0)	37 (26.6)	0.53, 0.97
Leukopenia	129 (46.7)	33 (12.0)	68 (48.9)	19 (13.7)	0.67, 0.62
Anemia	117 (42.4)	52 (18.8)	51 (36.7)	23 (16.5)	0.26, 0.57
Decreased lymphocyte count	114 (41.3)	54 (19.6)	52 (37.4)	20 (14.4)	0.44, 0.19
Decreased platelet count	81 (29.3)	34 (12.3)	43 (30.9)	23 (16.5)	0.74, 0.24
Increased neutrophil count	58 (21.0)	1 (0.4)	23 (16.5)	0	0.28, 0.48
Increased white cell count	45 (16.3)	4 (1.4)	17 (12.2)	2 (1.4)	0.27, 0.99
Increased monocyte cell count	42 (15.2)	0	7 (5.0)	0	0.002, NA

Data are number of patients (%)

*P*_*1*_* P*-value of any grade, *P*_*2*_* P*-value of grade 3 or 4, *NA* Not available

The safety population included all patients who received at least one dose of the study drug. The listed adverse events of any grade are those that occurred in 15% or more of the patients in either group. The listed grade 3 or 4 adverse events are those that occurred in 5% or more of the patients in either group

The incidence of AEs leading to treatment termination were similar between the two groups (8.7% vs. 7.2%), and the most common AE was infectious pneumonia (1.8% vs. 3.6%). All of the AEs were transient and reversible.

SAEs were reported in 112 (40.6%) of 276 patients in the aponermin group and 52 (37.4%) of 139 patients in the placebo group. Pneumonia was the most common SAE (20.3% vs. 20.9%). SAEs occurred in 1% or more of the patients in either group are shown in Table S2.

### HRQoL assessment

The mean scores for each domain of the EORTC QLQ-C30 and MY20 at baseline have no difference between the two groups (Supplementary file: Table S3). The Least-Squares (LS) mean changes in scores from baseline over the treatment cycles showed that the difference between groups favored the aponermin group over the placebo group for global health status, emotional functioning, social functioning, fatigue, constipation, and financial difficulties in the QLQ-C30 (all *P*-values < 0.05). For other domains, no significant differences between groups were observed. Future perspective, body image, and disease symptoms of QLQ-MY20 in the aponermin group were significantly better than those in the placebo group (all *P*-values < 0.05). The LS mean changes for disease symptoms and side effects of treatment were stable across treatments in the aponermin group and was not worse than that in the placebo group. (Supplementary file: Table S4).

## Discussion

In this study, aponermin was combined with TD regimens. In China, thalidomide was more widely used than lenalidomide and bortezomib because of its affordability. Although a few new drugs for RRMM have been approved in China in the last five years, they are expensive, and some patients are deterred. Therefore, thalidomide remains an indispensable anti-myeloma drug. The superiority of aponermin plus TD over placebo plus TD in RRMM was confirmed in this study. The PFS, OS, and ORR were significantly improved in the aponermin group compared to the placebo group. The OS benefit of aponermin group vs. placebo group was further improved in the updated analysis than that in the first analysis, from 4.8 months to 6.0 months. In the aponermin group, more patients achieved deep remission, with the much higher rate of VGPR or better response compared to the placebo group. For patients who had achieved PR or better responses, greater benefits in PFS and OS were observed in the aponermin group vs. the placebo group, suggesting that patients who received aponermin treatment were able to maintain longer periods of remission and longer overall survival.

The benefits of the aponermin group regarding PFS and OS were observed in most prespecified subgroups, including those with poor prognosis, such as patients aged ≥ 65 years, previous exposure to TD/RD, refractory to PI and IMiD, or previous treated with more than three regimens. It is noteworthy that in patients with previous exposure to PI and IMiD, the risk of progression or death reduced by 45% and the risk of death reduced by 39% in the aponermin group compared to the placebo group. Of these, more than 50% patints had been treated with at least four regimens, and 38.3% had received lenalidomide. In the aponermin group, four patients had exposed to carfilzomib, bortezomib, and IMiD, one VGPR, one MR and two stable disease (SD) were observed; two patients had previously used monoclonal anti-CD38 antibody, bortezomib, and lenalidomide, one PR and one SD were obtained. The result suggests that aponermin combined with TD may be still an option even for patients with previous heavy treatment.

Overall, the efficacy outcomes in this study were consistent with the results of the phase 2 trial, in which improvements in PFS and ORR were observed in patients of the aponermin plus TD group compared with those in the TD group [22]. The safety profile in the study was also consistent with previous studies, with hepatotoxicity as the major adverse reaction of aponermin [18, 19, 22].

Treatment with aponermin may cause hepatotoxicity in some patients, as indicated by the elevated ALT and AST levels. The elevations of ALT and AST generally occurred after two days of treatment, reached a peak value after five days treatment of aponermin, and returned to normal or baseline levels before the next treatment cycle (the representative shown in Supplementary file: Figure S2A). No TBIL abnormalities accompanied by elevated ALT levels were observed. It is worth noting that approximately 28% of the patients in the aponermin group had an early transient elevation of the AST level on the second day of the first cycle, even reaching grade 3 or above. However, concurrently, the ALT level was not elevated or only slightly elevated (7% of patients) (the representative shown in Supplementary file: Figure S2B). The vast majority of these elevations were only detected in the first cycle and recovered quickly and spontaneously even though aponermin was not stopped. It is speculated that this transient elevation of AST may be associated with tumor lysis but not hepatotoxicity [18, 19, 22, 25].

The incidences of anemia and decreased lymphocyte count in the aponermin group were higher than that in the placebo group, but there was no statistical significance. After adjustment for drug exposure, rate of anemia was slightly lower in the aponermin group (132 vs. 140 events per 100 patient-years), and rates of decreased lymphocyte count were similar in the two groups (173 vs. 169 events per 100 patient-years). There was no decrease in leukocyte, platelet and neutrophil. The result suggests that aponermin has no hematological toxicity.

Pyrexia (grade 1/2) is a confirmed adverse reaction of aponermin, which was reported by 20.7% and 12.2% patients in the aponermin group and the placebo group, respectively. More patients reported positive urine leukocyte (15.9% vs. 8.6%) and increased monocyte cell count (15.2% vs. 5.0%) in aponermin group compared to the placebo group, but there was no difference in the laboratory test results between the two groups. Hypocalcemia, upper respiratory tract infections, hypertriglyceridemia and hyperglycemia are the known adverse reactions of dexamethasone. Increased incidences of these adverse events were observed in the aponermin group compare to the placebo group, which mainly related to the longer drug exposure (5 vs. 3 cycles). More patients with a history of diabetes (14.9% vs. 10.8%) may be another reason for the higher incidence of hyperglycemia. The higher incidence of hypocalcemia in aponermin group may also be related to tumor lysis caused by aponermin. Hypocalcemia, upper respiratory tract infections, hypertriglyceridemia and hyperglycemia have not been observed eigher in the preclinical study, or in clinical studies of aponermin monotherapy. Further researches are needed to determine whether the added of aponermin to TD will increase the risks.

There was no evidence that aponermin leads to nephrotoxicity, cardiotoxicity, or secondary tumors. The incidence of SAEs was similar between the two groups.

The clinical benefit of aponermin was further supported by the results of HRQoL. In half of the domains of the QLQ-C30 and MY20, the LS mean changes of the aponermin group were significantly better compared to the placebo group. Disease symptoms and side effects of treatment of the QLQ-MY20 were stable across treatments in the aponermin group and were not worse than those in the placebo group. The result suggests that aponermin owned a good safety profile in clinic, and there is a broad space for its combined application with other antitumor drugs.

In this study, efficacy analyses were based on a mITT population, with two patients excluded from the analysis for not receiving any study treatment. Sensitivity analysis of PFS for the mITT population was performed. The results of intention-to-treat population (417 patients) (median PFS 5.5 months for the aponermin group and 3.1 months for the placebo group, HR, 0.62; 95% CI, 0.49–0.78; *P* < 0.0001) were completely consistent with those of the mITT population.

The main limitation of this study is that the efficacy outcomes for both the aponermin group and the placebo groups were slightly weaker compared with the triplet regimens of novel drugs approved in recent years. In particular, the ORR of the placebo group was only 13.7%, which was lower than previously reported [26–28]. However, cross-trial comparisons are confounded by differences in patients populations and study designs. In this study, 46.0% patients had received at least four regimens, 73.6% had received PI, 86.6%, IMiD. Importantly, 74.6% of the patients had previously exposed to TD/RD treatment (no documented refractoriness to TD/RD regimens). Due to the above reasons, TD regimen of the placebo group showed weak efficacy in this trial. Although there was a significant improvement when adding aponermin to the TD regimen, the improvement was limited. In order to get better clinical benefit, it will be important to improve the overall response rate based on the application of biomarkers and combination with more potent anti-myeloma drugs. As a next step, we will design clinical trials using aponermin plus bortezomib/carfilzomib, lenalidomide/pomalidomide, or CD38-targeting antibody for the treatment of RRMM.

Overall, the results of this study indicate that aponermin plus TD had a favourable benefit-risk profile compared with placebo plus TD in RRMM. The role of the TRAIL signalling pathway in inducing apoptosis has been explored for a long time. But, at present, no drug have been approved for anti-tumor therapy targeting death receptors 4/5. To our knowledge, this is the first phase 3 trial shows that activation of the TRAIL pathway is a feasible approach for the treatment of RRMM. This represents a genuine breakthrough in cancer treatment and brings a novel weapon to the arsenal for fighting cancers. Additionally, this opens the door to exploring the applications of TRAIL family members in other cancers.

In conclusion, this phase 3 study demonstrated that aponermin plus TD has a favorable benefit-risk profile compared with placebo plus TD. Aponermin plus TD significantly improved PFS, OS, and ORR with manageable and reversible toxicity in RRMM patients with at least two prior therapies, and should be considered an effective treatment option for RRMM patients by targeting death receptors 4/5.

### Supplementary Information

**Additional file 1.**

## Data Availability

The data that support the findings of this study are available upon reasonable request, by contact 13,381,075,598@163.com.

## Abbreviations

AE: Adverse event
ALT: Alanine transaminase
AST: Aspartate transaminase
CI: Confidence interval
CPT: Recombinant circularly permuted human TRAIL
CR: Complete response
DOR: Duration of response
DR4/5: Death receptor 4 or 5
EORTC: European Organization for Research and Treatment of Cancer
HR: Hazard ratio
HRQoL: Health-related quality of life
IAC: Independent assessment committee
IMiD: Immunomodulator
IMWG criteria: International Myeloma Working Group consensus criteria
ISS: International Staging System
IWRS: Interactive web response system
LDH: Lactate dehydrogenase
LS: Least-Squares
mITT: Modified intention-to-treat population
MM: Multiple myeloma
ORR: Overall response rate
OS: Overall survival
PD: Progressive disease
PFS: Progression-free survival
PI: Proteasome inhibitor
RD: Lenalidomide plus dexamethasone
RRMM: Relapsed or refractory multiple myeloma
SAEs: Serious adverse events
SD: Stable disease
TBIL: Total bilirubin
TD: Thalidomide plus dexamethasone
TRAIL: Tumor necrosis factor-related apoptosis-inducing ligand
TTP: Time to progression
TTR: Time to response
ULN: Upper limit of normal
VGPR: Very good partial response
