# Supplementary material for: Aponermin or placebo in combination with thalidomide and dexamethasone in the treatment of relapsed or refractory multiple myeloma (CPT-MM301): a randomised, double-blinded, placebo-controlled, phase 3 trial
Source: BMC Cancer. 2023 Oct 14;23:980. doi: 10.1186/s12885-023-11489-8 (PMC10576321; doi:10.1186/s12885-023-11489-8)
Supplement: Supplementary file 1 — Additional file 1. [file 12885_2023_11489_MOESM1_ESM.docx]

Supplementary file

Zhongjun Xia, Yun Leng, Baijun Fang, et al. Aponermin or placebo in combination with thalidomide and dexamethasone in the treatment of relapsed or refractory multiple myeloma (CPT-MM301): a randomised, double-blinded, placebo-controlled, phase 3 trial.

Table of Contents

[LIST OF INVESTIGATORS 2](#_Toc131087777)

[ELIGIBILITY CRITERIA 4](#_Toc131087778)

[SUPPLEMENTARY RESULTS 6](#_Toc131087779)

[Figure S1 Analysis of Overall Survival in modified Intention-to-Treat Population 6](#_Toc131087780)

[Figure S2. Representative alterations of serum TBIL, ALT, and AST in patients treated with aponermin, thalidomide and dexamethasone 8](#_Toc131087781)

[Table S1. Summary of the Efficacy Results Assessed by Investigators in modified Intention-to-Treat Population 9](#_Toc131087782)

[Table S2. Serious Adverse Events 10](#_Toc131087783)

[Table S3. Mean scores of EORTC QLQ-C30 and QLQ-MY20 at Baseline 11](#_Toc131087784)

[Table S4. Score changes from baseline in EORTC QLQ-C30 and QLQ-MY20 12](#_Toc131087785)

# LIST OF INVESTIGATORS

Zhongjun Xia, M.D. and Yang Liang, M.D. Department of Hematologic Oncology, Sun Yat-sen University Cancer Center, Guangzhou

Wenming Chen, M.D., Ph.D., Yun Leng, M.D., Zhongxia Huang, M.D. and Xin Li, M.D. Department of Hematology, Beijing Chao-Yang Hospital Capital Medical University, Beijing

Baijun Fang, M.D., Ph.D. and Yuzhang Liu, M.D. Department of Hematology, Henan Cancer Hospital, Henan Cancer Hospital Affiliated to Zhengzhou University, Zhengzhou

Wei Li, Ph.D. and Sujun Gao, M.D. Department of Hematology, The First Hospital of Jilin University, Changchun

Chengcheng Fu, Ph.D. and Lingzhi Yan, M.D., Ph.D. Department of Hematology, The First Affiliated Hospital of Soochow University, Jiangsu Institute of Hematology National Clinical Research Center for Hematologic Diseases, Suzhou

Linhua Yang, Ph.D. and Yanping Ma, Ph.D. Department of Hematology, Second Hospital of Shanxi Medical University, Taiyuan

Xiaoyan Ke, M.D. and Hongmei Jing, M.D. Department of Hematology and Lymphoma Research Center, Peking University Third Hospital, Beijing

Hua Jiang, M.D. and Juan Du, M.D., Ph.D. Department of Hematology, Changzheng Hospital, Second Military Medical University, Shanghai

Jianyu Weng, Ph.D. and Wei Ling, M.D. Department of Hematology, Guangdong Provincial People's Hospital, Guangdong Academy of Medical Sciences, Guangzhou

Li Liu, M.D. and Jingyi Zhang, M.D. Department of Hematology, Tangdu Hospital, Fourth Military Medical University, Xi'an

Yaozhong Zhao, B.S. and Weiwei Sui, M.D. Institute of Hematology & Blood Diseases Hospital, Chinese Academy of Medical Sciences & Peking Union Medical College, Tianjin

Xuejun Zhang, M.D. and Fuxu Wang, M.D. Department of Hematology, The Second Hospital of Hebei Medical University, Shijiazhuang

Aichun Liu, Ph.D. and Chuiming Jia, M.D. Department of Hematology, Harbin Medical University Cancer Hospital, Harbin

Qingzhi Shi, B.S. and Li Yu, M.D. Department of Hematology, The Second Affiliated Hospital of Nanchang University, Nanchang

Yuhuan Gao, M.D. and Lihong Liu, M.D. Department of Hematology, Fourth Hospital of Hebei Medical University, Shijiazhuang

Xiequn Chen, Ph.D. and Guangxun Gao, M.D. Department of Hematology, XiJing Hospital, Fourth Military Medical University, Xi’an

Ling Pan, Ph.D. and Ting Niu, Ph.D. Department of Hematology, West China Hospital, Sichuan University, Chengdu

Zhen Cai, M.D., Ph.D. and Donghua He, M.D. Bone Marrow Transplantation Center, The First Affiliated Hospital, School of Medicine, Zhejiang University, Hangzhou

Zhao Wang, Ph.D. and Na Wei, M.D. Department of Hematology, Beijing Friendship Hospital, Capital Medical University, Beijing

Yafei Wang, M.D. and Xiaofang Wang, M.D. Department of Hematology, Tianjin Medical University Cancer Institute and Hospital, Tianjin

Yaqun Fan, M.D. and Bing Xu, M.D. Department of Hematology, The First Affiliated Hospital of Xiamen University and Institute of Hematology, Medical College of Xiamen University, Xiamen

Ming Hou, Ph.D. and Luqun Wang, M.D. Department of Hematology, Qilu Hospital of Shandong University, Jinan

Yigai Ma, B.S. and Zhenling Li, M.D. Department of Hematology, China-Japan Friendship Hospital, Beijing

Jianda Hu, M.D., Ph.D. and Tingbo Liu, M.D. Fujian Institute of Hematology, Fujian Province Key Laboratory of Hematology, Fujian Medical University Union Hospital, Fuzhou

Jing Liu, Ph.D. and Xin Li, M.D. Department of Hematology, The Third Xiangya Hospital of Central South University, Changsha

Jianfeng Zhou, M.D., Ph.D., and Dengju Li, M.D. Department of Hematology, Tongji Hospital of Tongji Medical College, Huazhong University of Science and Technology, Wuhan

Xiaohong Zhang, M.D. and Xian Li, M.D. Department of Hematology, The Second Affiliated Hospital of Zhejiang University School of Medicine, Hangzhou

Haitao Meng, M.D., Ph.D. and Liangshun You, M.D. Department of Hematology, The First Affiliated Hospital, School of Medicine, Zhejiang University, Hangzhou

Xuzhang Lu, Ph.D. and Wenmin Han, M.D. Department of Hematology, The Affiliated Changzhou No.2 People’s Hospital of Nanjing Medical University, Changzhou

Fei Li, M.D. and Fancong Kong, M.D. Department of Hematology, First Affiliated Hospital of Nanchang University, Nanchang

Hanyun Ren, M.D., Ph.D. and Yujun Dong, M.D. Department of Hematology, Peking University First Hospital, Beijing

Bintao Huang, Ph.D. and Da Gao, M.D. Department of Hematology, The Affiliated Hospital of Inner Mongolia Medical University, Hohhot

Zonghong Shao, Ph.D. and Yihao Wang, M.D. Department of Hematology, General Hospital of Tianjin Medical University, Tianjin

Hebing Zhou, M.D. and Yong Zhang, M.D. Department of Hematology, Beijing Luhe Hospital, Capital Medical University, Beijing

Yu Hu, Ph.D. and Chunyan Sun, M.D., Ph.D. Department of Hematology, Union Hospital, Tongji Medical College, Huazhong University of Science and Technology, Wunan

All the investigators are in China.

# ELIGIBILITY CRITERIA

| Inclusion Criteria | Exclusion Criteria |
| --- | --- |
| 1.Sign informed consent form (ICF).  2.Age ≥ 18 and ≤ 75 years old when signing ICF.  3.Patients with RRMM who have received at least 2 treatment regimens for multiple myeloma (MM), and who do not consider bone marrow transplantation.  4.During the screening phase of the study, myeloma M protein (obtained by serum/urine protein electrophoresis) reaches a measurable level, and must meet at least one of the following detection indicators:  •Serum M protein ≥ 10 g/L (protein electrophoresis method) or IgA ≥ 7.5 g/L (turbidimetric immunoglobulin quantification).  • Urine M protein ≥ 0.2 g/24 hours.  5.Eastern Cooperative Oncology Group (ECOG) score ≤ 2.  6.The expected survival time is longer than 4 months.  7.Laboratory test results should meet the following specified indicators:  •Absolute neutrophil count (ANC) ≥ 1.0×10^9^/L.  •Platelet (PLT) ≥ 50×10^9^/L.  •Aspartate transaminase (AST) ≤ 2.5 ×ULN.  •Alanine transaminase (ALT) ≤ 2.5×ULN.  •Alkaline phosphatase (ALP) ≤ 2.5×ULN.  •Total bilirubin (TBIL) ≤ 1.5×ULN.  •Creatinine clearance rate (Ccr) ≥ 30 ml/min.  8.Comply with the relevant provisions of the ‘Management plan of prevention of pregnancy risk of thalidomide’. | 1.Stem cell transplant recipients with graft-versus-host disease that is active or requires immunosuppressive therapy when signing the ICF.  2.Patients who are refractory to TD or TD-containing regimens: patients who after the most recent treatment with TD or TD-containing regimens (≥ 2 courses and dexamethasone dosage ≥ 120 mg/treatment course) meet one of the following conditions:  •The patient did not achieve minimal response (MR) as the best efficacy.  •Disease progressed during treatment.  •Disease progressed within 60 days after the last medication.  3.Patients who are refractory to RD or RD-containing regimens: patients who after the most recent treatment with RD or RD-containing regimens (≥ 2 courses and dexamethasone dosage ≥ 120 mg/treatment course) meet one of the following conditions:  •The patient did not achieve MR as the best efficacy.  •Disease progressed during treatment.  •Disease progressed within 60 days after the last medication.  4.Patients who are allergic or intolerant to thalidomide or dexamethasone.  5.Patients who received any conventional or investigational anti-MM drug treatment (drug specifications indicate that the drug is used for anti-MM treatment; the limitation for thalidomide maintenance treatment can be reduced to within 2 weeks before the trial) or radiotherapy (limitation for restricted field radiotherapy can be reduced to within 2 weeks before the trial) within 4 weeks before the trial.  6.Patients who received major surgeries within the past 2 weeks.  7.Patients who have participated in aponermin clinical trials in the past.  8.Patients with plasma cell leukemia.  9.Patients combined with other malignant tumors.  10.Patients with poorly controlled hypertension or clinically significant (for example, active) cardiovascular and cerebrovascular diseases, such as cerebrovascular accidents (within 6 months before signing the ICF), myocardial infarction (within 6 months before signing the ICF), unstable angina, congestive heart failure grade II or above by the New York Heart Association classification, or severe arrhythmia that is poorly controlled or may have potential impact on the treatment of the study.  11. Patients combined with other serious organic diseases and mental illnesses.  12. Patients with systemic active infections that need treatment.  13. Known Human Immunodeficiency Virus (HIV)-positive patients or patients with clinically active hepatitis A, B, or C.  14. Pregnant or breastfeeding female. |

# SUPPLEMENTARY RESULTS

# Figure S1 Analysis of Overall Survival in modified Intention-to-Treat Population

**A.**


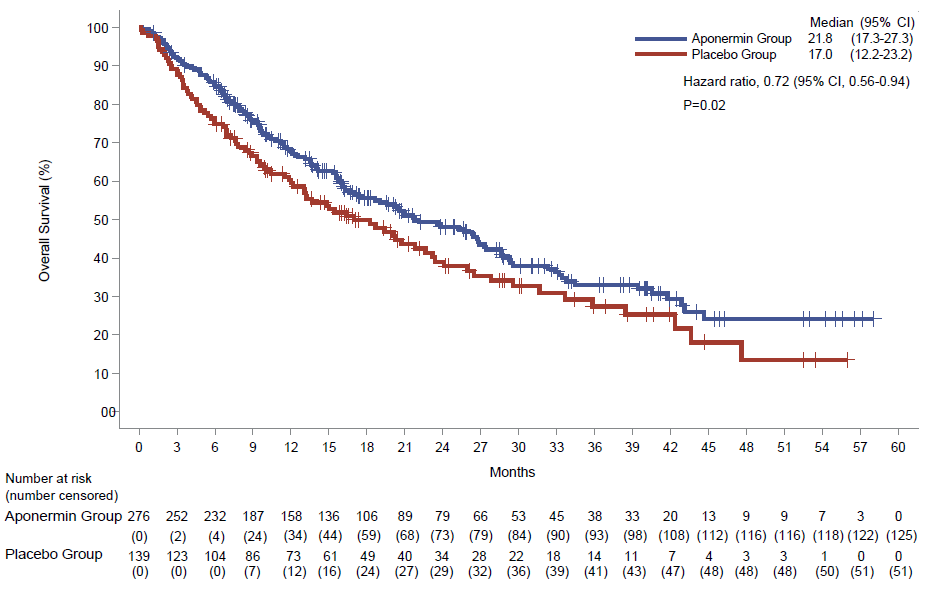


**B.**


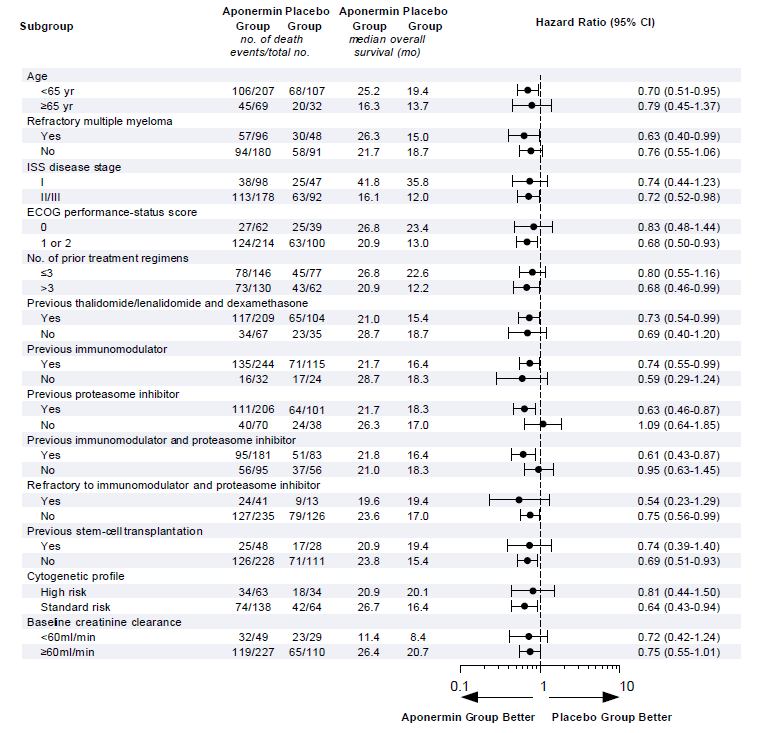


**Panel A.** **The results of the Kaplan-Meier analysis of overall survival in the modified Intention-to-Treat Population. Panel B. The results of subgroup analysis of overall survival.** In the analysis of overall survival with a median follow-up of 30.1 months (95% CI, 25.9–34.0), 4.8 months extension was observed in the aponermin group compared to that in the placebo group. The pre-specified subgroup analysis showed that the effect of aponermin on overall survival compared to placebo was consistent for most subgroups.

# Figure S2. Representative alterations of serum TBIL, ALT, and AST in patients treated with aponermin, thalidomide and dexamethasone

**A.**


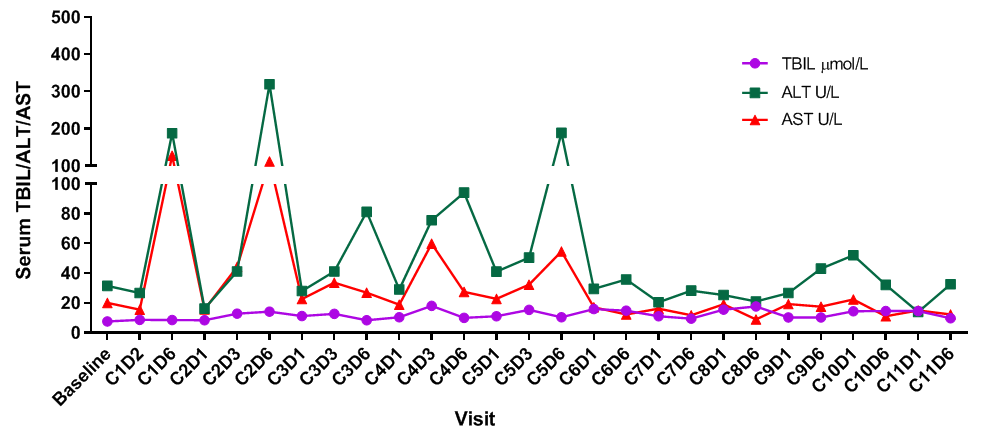


**B.**


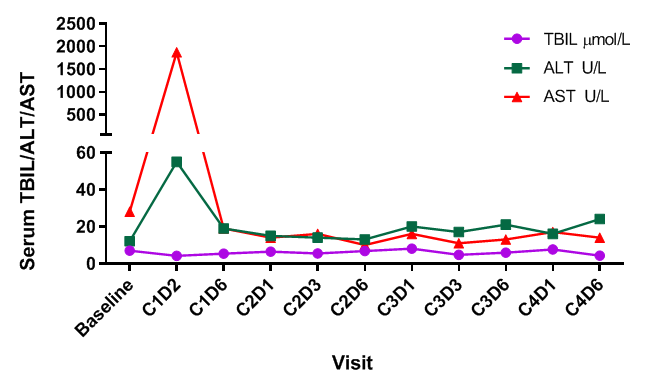


**Panel A. The characteristics of ALT, AST, and TBIL alterations in representative patient with hepatotoxicity after aponermin treatment. Panel B. The characteristics of transient elevation of AST on the second day of the first course of treatment (C1D2) in representative patient after single dose treatment of aponermin, which may be associated with tumor lysis but not hepatotoxicity.** Aponermin was administered via intravenous infusion on days 1 to 5 of each 28-day cycle. Thalidomide was given orally once daily. Dexamethasone was given orally on days 1 to 4.

Serum samples were collected prior to aponermin dosing (day 1 of cycle x, CxD1) and on day 2 of cycle 1 (C1D2), day 3 of cycle x (CxD3) (except cycle 1) and day 6 of cycle x (CxD6) of each cycle for TBIL, ALT and AST tests.

Abbreviations: TBIL, total bilirubin; ALT, alanine transaminase; AST, aspartate transaminase.

# Table S1. Summary of the Efficacy Results Assessed by Investigators in modified Intention-to-Treat Population

|  | **Aponermin Group**  **(N=276)** | **Placebo Group**  **(N=139)** | **Proportion difference between groups** | **Hazard Ratio (95%CI)** | **P value** |
| --- | --- | --- | --- | --- | --- |
| Overall response — % (95% CI) | 30.4 (25.1–36.2) | 10.8 (6.2–17.2) | 19.6 (12.2–27.1) | ·· | <0.001 |
| Clinical benefit — % (95% CI)* | 44.9 (39.0–51.0) | 29.5 (22.1–37.8) | 15.4 (5.8–25.0) | ·· | 0.003 |
| Very good partial response or better— % (95% CI) | 13.4 (9.6–18.0) | 2.2 (0.4–6.2) | 11.2 (6.6–15.9) | ·· | 0.001 |
| Best response — % (95% CI)^†^ |  |  |  |  |  |
| Stringent complete response | 1.8 (0.6–4.2) | 0.7 (0.0–3.9) | 1.1 (-1.0–3.2) | ·· | 0.67 |
| Complete response | 0 (0–1.3) | 0 (0–2.6) | ·· | ·· | ·· |
| Very good partial response | 11.6 (8.1–16.0) | 1.4 (0.2–5.1) | 10.2 (5.9–14.4) | ·· | <0.001 |
| Partial response | 17.0 (12.8–22.0) | 8.6 (4.5–14.6) | 8.4 (2.0–14.8) | ·· | 0.03 |
| Minimal response^‡^ | 14.5 (10.6–19.2) | 18.7 (12.6–26.2) | -4.2 (-11.9–3.5) | ·· | 0.32 |
| Stable disease | 37.0 (31.2–42.9) | 43.9 (35.5–52.5) | -6.9 (-17.0–3.1) | ·· | 0.20 |
| Progression | 13.4 (9.6–18.0) | 24.5 (17.6–32.5) | -11.1 (-19.3–-2.9) | ·· | 0.006 |
| Not evaluable | 4.7 (2.5–7.9) | 2.2 (0.4–6.2) | 2.6 (-0.9-6.0) | ·· | 0.28 |
| Median Progression-free survival — mo (95% CI) | 4.8 (3.9–6.1) | 3.5 (2.2–3.9) | ·· | 0.66 (0.52–0.83) | <0.001 |
| Median time to progression — mo (95% CI) | 5.4 (4.6–6.6) | 3.7 (2.4–4.4) | ·· | 0.66 (0.52– 0.84) | 0.001 |

^*^A clinical benefit was defined as a minimal response (MR) or better.

^†^The best confirmed responses were assessed by investigators in a blinded manner according to the International Myeloma Working Group criteria.

^‡^Minimal response was assessed according to the European Group for Blood and Bone Marrow Transplant criteria.

# Table S2. Serious Adverse Events

|  | Aponermin Group (N=276) | Placebo Group (N=139) |
| --- | --- | --- |
| Any serious adverse event | 112 (40.6) | 52 (37.4) |
| Pneumonia | 56 (20.3) | 29 (20.9) |
| Death | 10 (3.6) | 2 (1.4) |
| Upper respiratory tract infection | 5 (1.8) | 3 (2.2) |
| Heart failure | 4 (1.4) | 5 (3.6) |
| Anemia | 4 (1.4) | 2 (1.4) |
| Drug-induced liver injury | 4 (1.4) | 0 |
| Gastroenteritis | 3 (1.1) | 1 (0.7) |
| Elevated alanine aminotransferase | 3 (1.1) | 0 |
| Respiratory tract infection | 3 (1.1) | 0 |
| Elevated aspartate aminotransferase | 3 (1.1) | 0 |
| Bronchitis | 3 (1.1) | 0 |
| Abnormal liver function | 3 (1.1) | 0 |
| Renal failure | 3 (1.1) | 0 |
| Tumor lytic syndrome | 3 (1.1) | 0 |
| Blurred vision | 3 (1.1) | 0 |
| Sepsis | 2 (0.7) | 2 (1.4) |
| Decreased platelet | 2 (0.7) | 2 (1.4) |
| Septic shock | 0 | 3 (2.2) |
| Pain | 0 | 2 (1.4) |

Shown are serious adverse events that occurred in 1% or more of the patients in either group.

# Table S3. Mean scores of EORTC QLQ-C30 and QLQ-MY20 at Baseline

|  | **Aponermin Group**  **(N=276)** | **Placebo Group**  **(N=139)** | **P value**^*^ |
| --- | --- | --- | --- |
| **QLQ-C30** |  |  |  |
| **Global health status/QoL** | 56.52 (23.700) | 56.81 (22.716) | 0.91 |
| **Function scales** |  |  |  |
| Physical functioning | 74.66 (20.489) | 74.49 (21.915) | 0.94 |
| Role functioning | 74.87 (26.995) | 73.20 (27.015) | 0.57 |
| Emotional functioning | 81.28 (18.706) | 82.12 (16.812) | 0.67 |
| Cognitive functioning | 79.54 (18.604) | 81.35 (19.161) | 0.38 |
| Social functioning | 62.28 (29.827) | 62.28 (29.827) | 0.77 |
| **Symptom scales** |  |  |  |
| Fatigue | 33.94 (20.594) | 33.42 (20.049) | 0.82 |
| Nausea and vomiting | 4.54 (12.360) | 4.89 (13.345) | 0.80 |
| Pain | 30.97 (27.300) | 27.65 (25.748) | 0.26 |
| Dyspnoea | 19.78 (22.376) | 19.51 (20.436) | 0.91 |
| Insomnia | 22.45 (26.023) | 22.49 (26.942) | 0.90 |
| Appetite loss | 14.09 (23.908) | 14.25 (20.015) | 0.95 |
| Constipation | 19.46 (26.953) | 19.24 (26.656) | 0.94 |
| Diarrhoea | 6.94 (14.533) | 5.29 (12.936) | 0.28 |
| Financial difficulties | 61.34 (33.997) | 60.00 (33.601) | 0.72 |
| **QLQ-MY20** |  |  |  |
| **Functional scales** |  |  |  |
| Future perspective | 64.52 (25.373) | 66.58 (23.412) | 0.45 |
| Body image | 66.67 (28.888) | 65.61 (28.577) | 0.74 |
| **Symptom scales** |  |  |  |
| Disease symptoms | 24.51 (20.671) | 22.03 (17.011) | 0.22 |
| Side effects of treatement | 21.75 (15.568) | 21.45 (12.928) | 0.84 |

^*^Group t-tests were used for assessing the differences between groups.

Data are mean (SD). The scores have been standardized by linear transformation. Higher scores for symptom items indicate greater symptom severity; higher scores for function and global health status/QoL items indicate better function and health status. QoL, quality of life.

# Table S4. Score changes from baseline in EORTC QLQ-C30 and QLQ-MY20

|  | **LS Mean Change (SE) [95% CI]** | | | **P value** |
| --- | --- | --- | --- | --- |
|  | **Aponermin Group(N=276)** | **Placebo Group(N=139)** | **Difference between groups** |  |
| **QLQ-C30** |  |  |  |  |
| **Global health status/QoL** | -1.0 (1.63) [-4.2–2.2] | -6.1 (2.31) [-10.7–-1.6] | 5.1 (2.48) [0.2–10.0] | 0.04 |
| **Function scales** |  |  |  |  |
| Physical functioning | -9.1 (1.52) [-12.1–-6.1] | -11.3 (2.15) [-15.5–-7.1] | 2.2 (2.31) [-2.3–6.8] | 0.34 |
| Role functioning | -16.6 (2.08) [-20.7–-12.5] | -20.0 (2.97) [-25.8–-14.1] | 3.4 (3.18) [-2.9–9.6] | 0.29 |
| Emotional functioning | -6.9 (1.49) [-9.8–-4.0] | -11.9 (2.12) [-16.1–-7.7] | 5.0 (2.27) [0.5–9.4] | 0.03 |
| Cognitive functioning | -10.0 (1.68) [-13.3–-6.7] | -14.9 (2.43) [-19.6–-10.1] | 4.9 (2.61) [-0.3–10.0] | 0.06 |
| Social functioning | -6.1 (2.10) [-10.2–-2.0] | -15.6 (3.03) [-21.6–-9.7] | 9.5 (3.26) [3.1–15.9] | 0.004 |
| **Symptom scales** |  |  |  |  |
| Fatigue | 4.5 (1.58) [1.4–7.6] | 9.3 (2.26) [4.9–13.7] | -4.8 (2.43) [-9.6–-0.0] | 0.049 |
| Nausea and vomiting | 1.2 (0.94) [-0.7–3.1] | 1.9 (1.38) [-0.8–4.7] | -0.7 (1.49) [-3.7–2.2] | 0.62 |
| Pain | 2.9 (1.78) [-0.6–6.4] | 6.3 (2.54) [1.3–11.3] | -3.4 (2.74) [-8.8–2.0] | 0.21 |
| Dyspnoea | 1.7 (1.78) [-1.8–5.2] | 3.9 (2.59) [-1.2–9.0] | -2.2 (2.80) [-7.7–3.3] | 0.44 |
| Insomnia | -2.7 (1.85) [-6.4–0.9] | 0.7 (2.65) [-4.5–6.0] | -3.5 (2.85) [-9.1–2.1] | 0.22 |
| Appetite loss | 4.7 (1.63) [1.5–7.9] | 8.1 (2.40) [3.4–12.8] | -3.4 (2.59) [-8.5–1.7] | 0.19 |
| Constipation | 10.5 (2.47) [5.7–15.4] | 20.7 (3.57) [13.7–27.7] | -10.2 (3.81) [-17.7–-2.7] | 0.008 |
| Diarrhoea | -2.1 (0.95) [-4.0–-0.2] | -2.0 (1.41) [-4.8–0.8] | -0.1 (1.55) [-3.1–2.9] | 0.95 |
| Financial difficulties | -9.1 (2.39) [-13.8–-4.4] | 2.6 (3.44) [-4.1–9.4] | -11.7 (3.70) [-19.0–-4.5] | 0.002 |
| **QLQ-MY20** |  |  |  |  |
| **Functional scales** |  |  |  |  |
| Future perspective | 1.9 (1.80) [-1.6–5.4] | -8.3 (2.56) [-13.4–-3.3] | 10.2 (2.75) [4.8–15.6] | 0.0002 |
| Body image | -6.3 (2.14) [-10.5–-2.1] | -20.8 (3.02) [-26.8–-14.9] | 14.5 (3.29) [8.1–21.0] | <0.0001 |
| **Symptom scales** |  |  |  |  |
| Disease symptoms | -2.4 (1.26) [-4.8–0.1] | 3.4 (1.79) [-0.1–6.9] | -5.7 (1.92) [-9.5–-2.0] | 0.003 |
| Side effects of treatement | 1.5 (1.11) [-0.7–3.7] | 3.6 (1.58) [0.5–6.7] | -2.1 (1.69) [-5.4–1.2] | 0.21 |

Least-Squares (LS) mean changes from baseline over treatment cycles were evaluated for each domain using mixed-model measure analysis, with treatment, baseline scores, strata variables, visit, and treatment-by-visit interaction as fixed effects and patients as a covariate. For global health status/QoL and function scales, a negative value indicates worsening from baseline; for symptom scales, a negative value indicates improvement from baseline. QoL, quality of life.
